# Exploring Prevalence Trends of Jaw Bone Pathologies: A Three-Year Institutional Study

**DOI:** 10.7759/cureus.60574

**Published:** 2024-05-18

**Authors:** Neha Kannan, Karthikeyan Ramalingam, Pratibha Ramani, Murugesan Krishnan

**Affiliations:** 1 Oral Pathology and Microbiology, Saveetha Dental College and Hospitals, Saveetha Institute of Medical and Technical Sciences, Saveetha University, Chennai, IND; 2 Oral and Maxillofacial Surgery, Saveetha Dental College and Hospitals, Saveetha Institute of Medical and Technical Sciences, Saveetha University, Chennai, IND

**Keywords:** mandible, maxilla, jaw bone, jaw lesions, osteomyelitis, mucormycosis, osteoradionecrosis, prevalence, maxillofacial infections, bone tumors

## Abstract

Background

Head and neck bone pathologies cover various conditions with diverse causes. Infections like osteomyelitis and dental abscesses can spread to soft tissues and bones, causing tissue death, inflammation, and systemic effects. Benign and malignant tumors can develop from soft tissue, cartilage, or bone, posing challenges for diagnosis and treatment. Studies on their prevalence in local populations are rare, obscuring our understanding of regional health dynamics.

Aim

In this study, we aimed to assess the prevalence of bone pathologies documented over the last three years from 2021 to 2023.

Materials and methods

Histopathologically confirmed cases of bone pathologies at Saveetha Dental College and Hospitals, Saveetha Institute of Medical and Technical Sciences, Saveetha University, Chennai, India, were gathered from the institutional database (DIAS: Dental Information Archiving Software) from January 1, 2021, to December 31, 2023. They were categorized into groups of infectious and inflammatory lesions, fibro-osseous lesions, malignancies originating from bone, malignancies invading bone, and miscellaneous conditions. The data was then compiled into a Google spreadsheet (Google, Inc., Mountain View, USA) for further analysis. Graphs were created to visualize the prevalence of bone pathologies enabling a descriptive exploration of temporal trends.

Results

A total of 2626 biopsy records were reviewed. Among these, 242 (9.21%) cases of bone-related pathologies were included, and the remaining 2384 (90.79%) entities without any mention of bone were excluded. Overall, considering all three years, 43.8% (100) bone-related lesions were reported in 2021, 30.3% (77) in 2022 and 25.9% (65) in the year 2023. Under each category, infectious and inflammatory lesions for 40.5% (98), fibro-osseous lesions for 14.9% (36), benign lesions for 2.9% (7), malignancies originating from bone for 1.7% (4), malignancies invading bone for 38% (93), and miscellaneous conditions for 1.65% (4) were reported. The highest number of infectious and inflammatory pathologies (53%) were reported in 2021. A steep fall was observed in 2022 and 2023 under the infectious and inflammatory category. The malignancies invading the bone showed almost similar distribution in all three years.

Conclusion

The observed variations highlight the unpredictability of bone pathologies, involving the jaw bones. We emphasize continuous observation and analysis to comprehend changing patterns in bone health.

## Introduction

The head and neck region, which contains an intricate web of bones, joints, muscles, nerves, and blood vessels, is an essential hub of human anatomy. This complex system synergistically coordinates several vital functions, including speech, gustation, mastication, perception, and facial expression. The head and neck region is also widely susceptible to bone diseases [1,2].

Head and neck bone pathologies encompass various conditions with different etiological reasons. Trauma to the head and neck can cause fractures, dislocations, and other traumatic injuries to the bones and joints, whether it comes from falls, sports injuries, or accidents. Osteoarthritis and degenerative disc disease are two examples of degenerative diseases that can cause gradual bone and cartilage degradation, which can result in pain, stiffness, and loss of function. From deep-seated osteomyelitis to dental abscesses, infections can penetrate the head and neck's soft tissues and bones, resulting in tissue necrosis, regional inflammation, and systemic consequences. Benign and malignant tumors can originate from soft tissue, cartilage, or bone, which presents difficulties for diagnosis and treatment [3] 

The most common benign bone pathology in the head and neck region in the Indian subcontinent is osteoma. It is an abnormal growth of mature cancellous or compact bone with a low Ki67 proliferative index. Osteomas are usually asymptomatic and are discovered incidentally during imaging for other purposes [4,5]. Osteosarcoma is the most prevalent, primary malignant bone pathology in the head and neck region and is known for its aggressive nature. Primitive bone-forming cells give rise to osteosarcoma. The long bones, such as the arms and legs, are typically affected, but they can also occur in the skull and jawbones of the head and neck [6,7]. Similarly, although uncommon, the head and neck area is susceptible to a wide range of bone abnormalities.

Identification of high-risk populations, elucidating underlying risk elements, and implementing targeted strategies for early identification and prevention all hinge on grasping the frequency of bone conditions in the head and neck [8,9]. Population-based studies, epidemiological surveys, and healthcare registries are vital tools for researchers to comprehensively explore the landscape of bone disorders within the head and neck region. By analyzing large datasets and examining trends over time, insights can be gained about the prevalence, distribution, and underlying factors contributing to these conditions. Such investigations enable the identification of at-risk populations and their demographic and geographic variations [8,10].

Thus through population-based studies, epidemiological surveys, and healthcare registries, researchers can glean valuable information regarding the prevalence and patterns of bone disorders affecting the head and neck region. Prevalence studies within regional populations are a rarity, their scarcity casting a veil over the understanding of local health dynamics [11]. This pioneering study thus aimed to assess the prevalence of bone pathologies documented over the last three years.

## Materials and methods

Histopathologically confirmed cases of various bone pathologies were systematically gathered from the institutional database, Dental Information Archival Software (DIAS), at Saveetha Dental College and Hospitals, Saveetha Institute of Medical and Technical Sciences, Saveetha University, Chennai, India. This comprehensive data retrieval process was conducted following the acquisition of ethical clearance from the institutional ethical board (IHEC/SDC/FACULTY/24/OPATH/027), ensuring adherence to international standards. 

The records, spanning three years from January 1, 2021 to December 31, 2023, were meticulously collected. This involved cross-referencing digital entries within the institution's database with manual records, ensuring the completeness and accuracy of the data. The search term "bone" was used to scan through all relevant bone-related pathologies and diseases entered into the software were methodically retrieved, providing a comprehensive overview of bone-related conditions within the institution. Following retrieval, cases of reported bone pathology were carefully isolated, excluding soft tissue tumors and non-bone-related conditions. To further enhance data integrity, digital records were cross-checked with manual registers to prevent missing any biopsy records, thereby ensuring the dataset's reliability for subsequent analysis and interpretation.

The identified lesions were subsequently categorized into distinct groups. These groups encompassed infectious and inflammatory lesions, fibro-osseous lesions, malignancies originating from bone, malignancies invading bone, and miscellaneous conditions. The pathologies under the Infectious and Inflammatory category included - fungal osteomyelitis including mucormycosis or aspergillosis, bacterial osteomyelitis, condensing osteitis, and necrotic bone; fibro-osseous lesions - ossifying fibroma, cemento-osseous dysplasia, and fibrous dysplasia; benign lesions - osteoma, enchondroma, osteochondroma, osteoblastoma, non-ossifying fibroma, chondroblastoma, chondromyxoid fibroma, and Langerhan cell histiocytosis; malignancies arising from the bone - osteosarcoma, chondrosarcoma, Ewing's sarcoma, and fibrosarcoma; malignancies invading the bone - squamous cell carcinoma. Miscellaneous conditions included diseases like Paget's disease, osteosclerosis, and other entities that did not fall under any of the above groups.

The clinical and demographic history was cross-checked for all the cases. Relevant clinical, radiological, and histopathological images were used for documentation and descriptive purposes. Subsequently, the pertinent data was entered into a Google spreadsheet (Google, Inc., Mountain View, USA) for analysis. Graphical representations were then generated to illustrate the prevalence of various bone pathologies observed in 2021, 2022, and 2023. This method facilitated a detailed exploration of temporal trends and fluctuations in the occurrence of bone pathologies and bone diseases within the specified timeframe through a descriptive analysis.

## Results

A total of 2626 biopsy records, from January 1, 2021, to December 31, 2023, were reviewed, including n = 242 (9.21%) cases of bone-related cases and excluding all the other entities without any mention of bone (2384 cases). The pathologies included mucormycosis, ossifying fibroma, cemento osseous dysplasia, condensing osteitis, osteomyelitis, Paget’s disease, osteosclerosis, osteoma, osteosarcoma, Langerhans cell histiocytosis, fibrous dysplasia, osteoblastoma, cherubism, necrotic bone, and malignancies invading bone.

**Figure 1:**
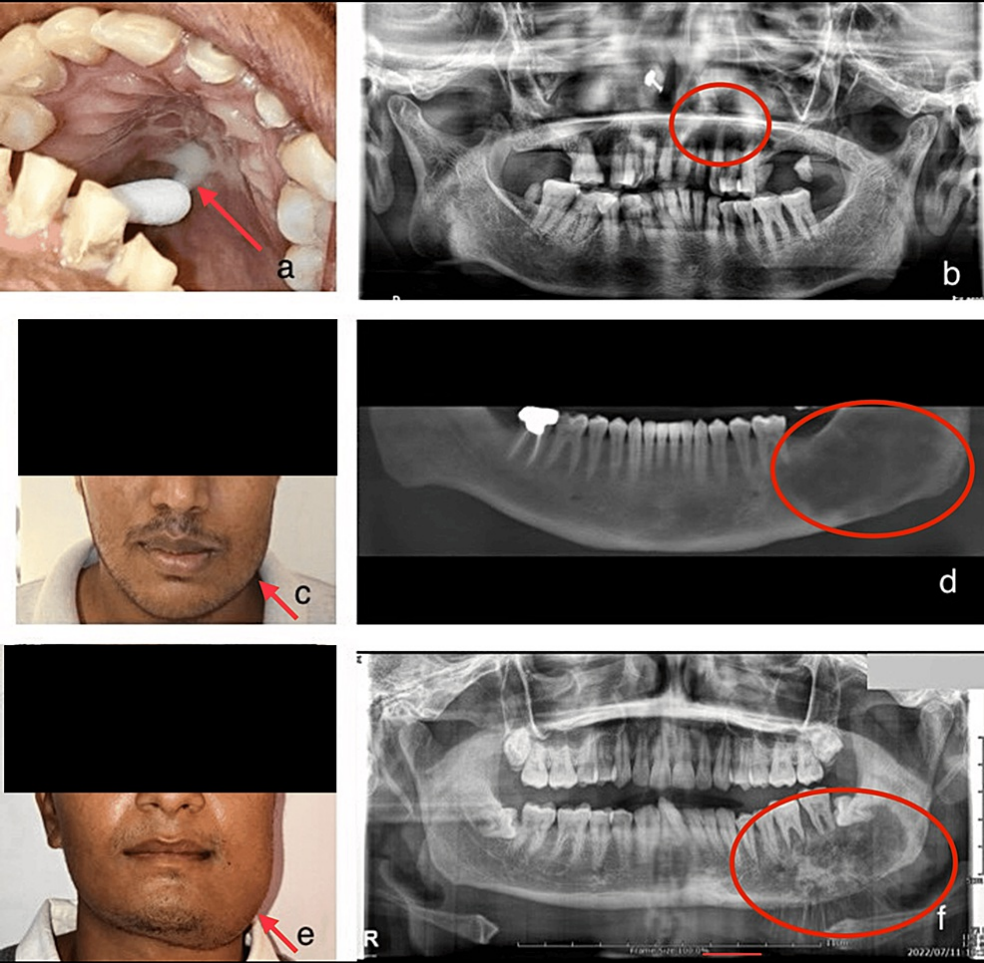
Figures showing clinical and radiographic images a) Purulent white discharge in the palatal region of fungal osteomyelitis - mucormycosis b) Orthopantomogram revealing a hazy radiolucency extending from 21 to 25 region in the maxilla of fungal osteomyelitis - mucormycosis c) Swelling involving the left lower side of the face - ossifying fibroma d) Cone beam computed tomography (CBCT) revealing a mixed lesion with indistinct borders extending from the distal part of 36 and extending posteriorly to the ramus of the mandible - ossifying fibroma e) Facial asymmetry involving the left lower part of the face - osteosarcoma f) Orthopantomogram revealing a typical sunburst appearance of a mixed osteolytic lesion on the left posterior mandible with indistinct borders - osteosarcoma

Relevant clinical details and radiological findings were retrieved from the DIAS for descriptive purposes (Figure 1).

**Figure 2:**
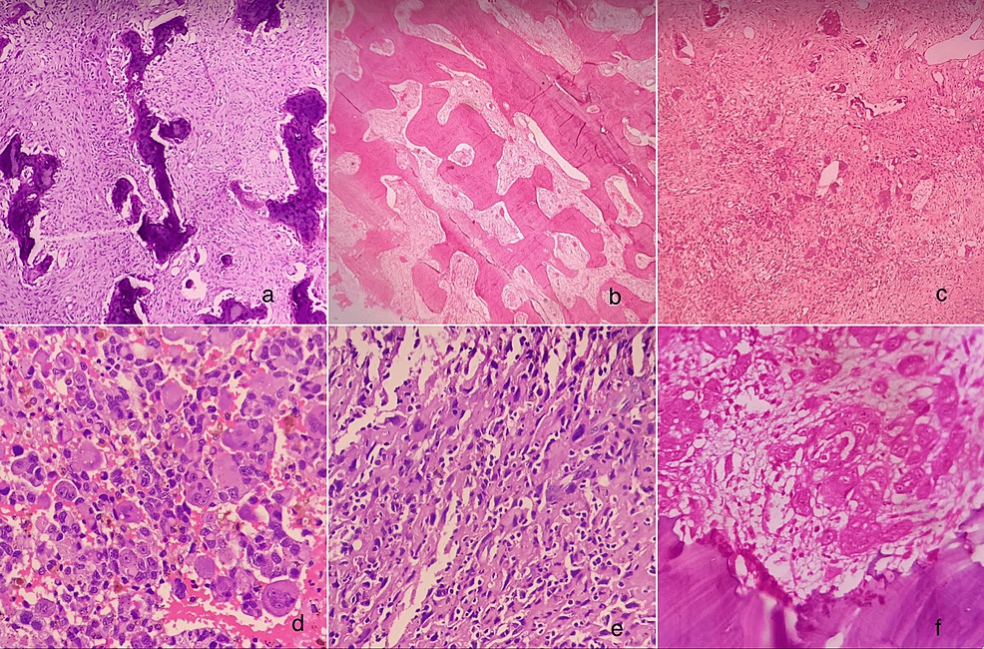
Photomicrographs a) Ossicles of bone within a cellular fibrous connective tissue stroma - ossifying fibroma (10x, H&E) b) Bony trabeculae in Chinese letter pattern - fibrous dysplasia (10x, H&E) c) Cellular connective tissue with bone elements and vascular channels - cherubism (4x, H&E) d) Sheets of malignant cells with areas of hemorrhage - osteosarcoma (40x, H&E) e) Diffuse proliferation of malignant cells - osteosarcoma (40x, H&E) f) Islands of malignant epithelial cells invading the bone - squamous cell carcinoma invading the bone (10x, H&E)

Histopathological findings were retrieved from the DIAS for descriptive purposes (Figure 2).

Under each category, infectious and inflammatory lesions, n = 98 (40.5%); fibro-osseous lesions, n = 36 (14.9%); benign lesions, n = 7 (2.9%); malignancies originating from bone, n = 4 (1.7%); malignancies invading bone, n = 93 (38%) were noted. All 93 cases were oral squamous cell carcinoma invading the adjacent jaw bones, and there were no cases of metastasis into the jaw bones. Miscellaneous conditions, n = 4 (1.65%) were reported (Table 1).

**Table 1:** Table showing bone pathologies observed in 2021, 2022, and 2023

List of bone pathologies	Cases reported in 2021	Cases reported in 2022	Cases reported in 2023
Mucormycosis	46	16	15
Ossifying fibroma	6	9	8
Cemento osseous dysplasia	3	4	1
Condensing osteitis	2	1	0
Osteomyelitis	3	5	4
Paget’s disease	1	1	0
Osteosclerosis	2	0	0
Osteoma	1	0	1
Osteosarcoma	1	2	1
Langerhans cell histiocytosis	1	0	1
Fibrous dysplasia	0	2	1
Osteoblastoma	1	2	0
Cherubism	0	0	2
Necrotic bone	2	2	2
Malignancies invading bone	31	33	29
Total cases	100	77	65

In 2021, a total of 100 cases of bone pathologies were identified among the 818 cases reported in total. This contributed to 12% of the reported cases. The most common bone pathology reported was fungal osteomyelitis - mucormycosis, n= 46 (46%). All 46 cases of mucormycosis were associated with COVID-19. Under each category, the highest reported cases were under the infectious and inflammatory category while the lowest reported were from the malignancies arising from the bone. Infectious and inflammatory conditions were the most prevalent, with 53 cases accounting for 53% of the total cases documented. Following this, fibro-osseous lesions constituted nine cases, representing 9% of the total, while benign pathologies accounted for six cases or 6%. Malignancies arising directly from bone were relatively rare, with only one case documented, constituting 1% of the total cases. In contrast, primary malignancies invading bone tissue were more prevalent, comprising 31 cases and constituting 31% of the total cases reported. Additionally, miscellaneous bone pathologies accounted for representing 4% of the total. 

The total number of bone-related cases reported in 2022 was 77 out of 894 (8.60%). The highest reported case was malignancies invading the bone with 33 cases documented, constituting 43% of the total cases reported. The second highest was fungal osteomyelitis - mucormycosis, n = 16 (21%), 10 of which were COVID-19-associated. Five patients had a history of diabetes while one patient had a history of chronic obstructive pulmonary disease. Similar to the year 2021, the least reported malignancies of the bone were n = 2 (2.6%). Infectious and inflammatory conditions were documented in 24 cases, constituting 31% of the cases observed. Fibro-osseous lesions were observed in 15 cases, comprising 19% of the total, while benign pathologies accounted for two cases (osteoblastoma), representing 2.6% of the total cases recorded. Malignancies arising from the bone were relatively rare, with only two cases of osteosarcoma, making up 2.60% of the total. In contrast, primary malignancies invading bone were more prevalent, There was only a single reported case under the miscellaneous category, contributing 1.3% to the overall total. 

The total number of reported bone pathologies in 2023 was 65 out of 914 records (7.10%). The highest number of cases were primary malignancies invading the bone with 29 cases documented, representing 44.6% of the total cases. Infectious and inflammatory conditions accounted for 21 cases, comprising 32%. Fibro-osseous lesions were identified in 12 cases, making up 18.5% of the total, while benign pathologies were present in two (Langerhans cell histiocytosis and osteoma), accounting for 3.07% of the recorded cases. Malignancies originating from bone were scarce, with only one reported case of osteosarcoma constituting 1.53% of the overall total. No instances of miscellaneous bone pathologies were noted in the dataset. 

In 2021, under the category of infectious and inflammatory lesions, the mean age of the patients affected was 48 years. Male patients were affected more than the females. Under fibro-osseous lesions, the mean age group was 40 years, and a female gender predilection. Malignancies originating from bone were seen in only one middle-aged male patient. Malignancies invading bone were common in the fifth decade among male patients. In 2022, under the category of infectious and inflammatory lesions, the mean age of the patients affected was 55 years. Male patients were affected more than the females. Under fibro-osseous lesions, the mean age group was 45 years, and equal gender predilection. Malignancies originating from bone were seen in two young male patients in their second decade. Malignancies invading bone were again frequent in the sixth decade among male patients. In 2023, the patient's mean age was 50-60 years under the infectious and inflammatory lesions. Male patients showed more prevalence when compared to female patients. Under fibro-osseous lesions, the mean age group was 50 years, and a female gender predilection. Malignancies originating from bone were seen in one middle-aged male patient. Malignancies infiltrating bone were more prevalent among male patients in their fifth to sixth decade (Table 2).

**Table 2:** Table showing the bone pathologies with mean age and gender distribution in 2021, 2022, and 2023

Year	Category	Mean age	Gender distribution
2021	Infectious and inflammatory lesions	48	Male predominance
	Fibro-osseous lesions	40	Female predilection
	Malignancies originating from bone	41	Male predominance
	Malignancies invading bone	54	Male predominance
2022	Infectious and inflammatory lesions	55	Male predominance
	Fibro-osseous lesions	45	Equal gender predilection
	Malignancies originating from bone	25	Male predominance
	Malignancies invading bone	65	Male predominance
2023	Infectious and inflammatory lesions	55	Male predominance
	Fibro-osseous lesions	50	Female predilection
	Malignancies originating from bone	41	Male predominance
	Malignancies invading bone	64	Male predominance

Table 1 presents the incidence of various bone-related conditions from 2021 to 2023. In 2021, a total of 818 biopsies were received, with 100 of them related to bone pathologies comprising 12%. In 2022, 894 biopsies were received of which 77 were bone pathologies comprising 8.6%. In 2023, 914 biopsies were received of which 65 were bone pathologies comprising 7.1%. The total number of biopsies received showed a slight increase over three years. However, the percentage of bone biopsies decreased gradually over the same period (Table 3).

**Table 3:** Table showing the total number of biopsies, bone biopsies, and their percentage

Year	Total number of biopsies	Bone pathologies	Percentage of bone pathologies
2021	818	100	12%
2022	894	77	8.6%
2023	914	65	7.1%

**Figure 3:**
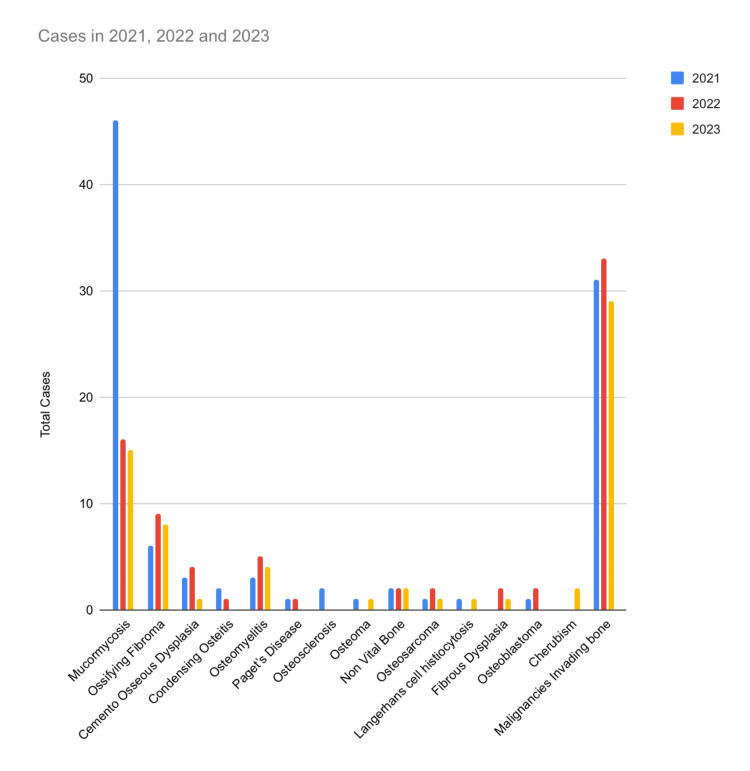
The graph presents cumulative data illustrating the trends in bone pathology spanning from 2021 to 2023

Among the listed conditions, mucormycosis exhibited a fluctuating trend, with 46 cases reported in 2021, followed by a decrease to 16 cases in 2022 and a further decrease to 15 cases in 2023. Other conditions, such as condensing osteitis, osteomyelitis, and Paget's disease, showed varied patterns of occurrence over three years. Notably, malignancies invading bone exhibited relatively consistent numbers, with 31, 33, and 29 cases reported in 2021, 2022, and 2023, respectively (Figure 3).

**Figure 4:**
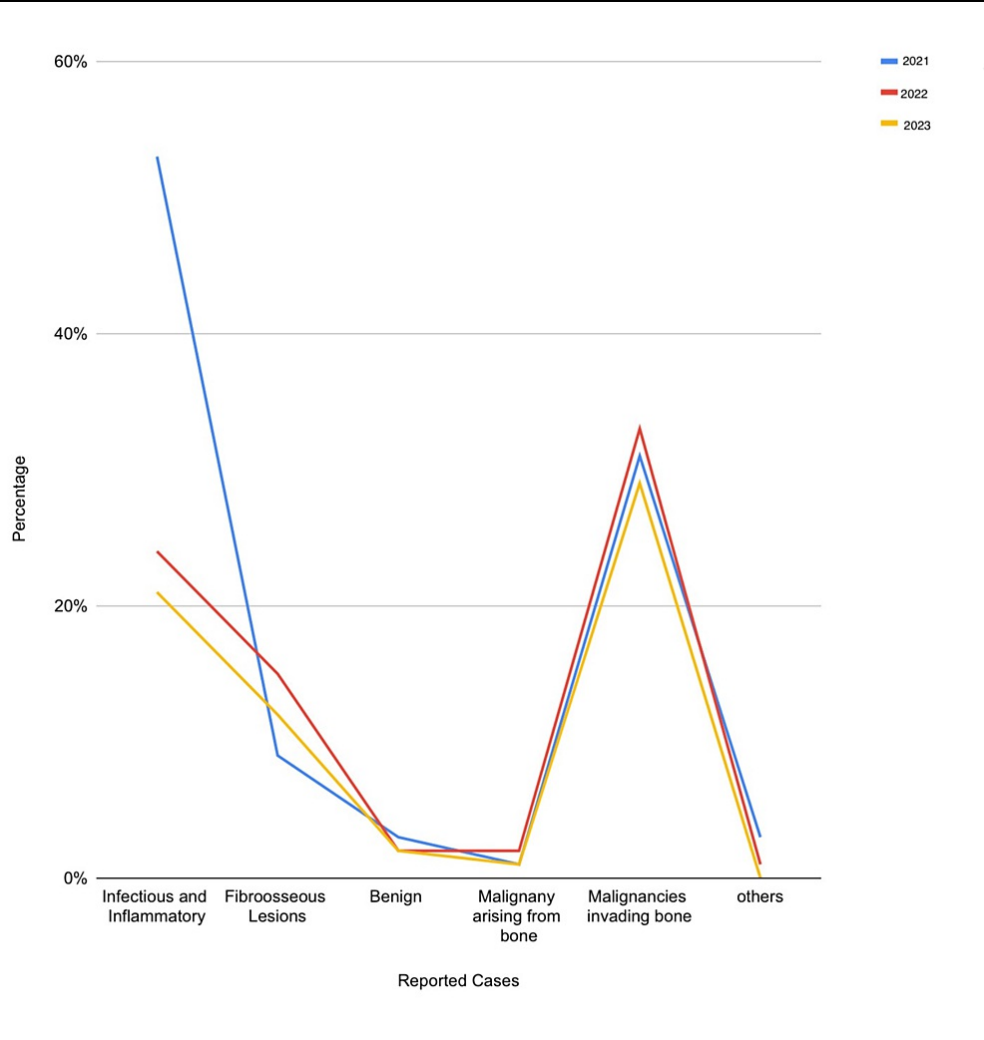
Line graph demonstrating the trends in bone pathology under different categories over three years

The highest number of infectious and inflammatory pathologies were reported in 2021 (53%). A steep fall is observed in the following years. The malignancies invading the bone show almost similar patterns in all three years (Figure 4).

## Discussion

Understanding the occurrence and trends of diverse bone disorders can be obtained through epidemiological investigations and healthcare repositories. This data could be the foundation for formulating various treatment strategies to meet patients' requirements and implementing preventive measures and early detection programs [10,12]. In the present study, the highest number of cases was reported in the year 2021 and the lowest was reported in the year 2023. The maximum reported case was fungal osteomyelitis - mucormycosis in the year 2021. Most of these cases were associated with a history of COVID-19. These findings were in concordance with an earlier investigation conducted by Mishra et al. in which they examined the clinical spectrum of COVID-associated mucormycosis (CAM) in individuals with diabetes and their subsequent outcomes [13]. It was discovered that out of 953 hospitalized individuals diagnosed with COVID-19 infection, 32 patients were identified to have CAM, resulting in an incidence rate of 3.36%. This could be attributed to the increased occurrence of mucormycosis in COVID-19-affected patients owing to many factors. Corticosteroids play a crucial role in treating COVID-19 patients requiring supplemental oxygen. While the standard practice involves administering prednisolone or an equivalent dosage of 1 mg/kg for over three weeks, this regimen is recognized as a risk factor for mucormycosis. Despite this, certain case reports have highlighted instances of mucormycosis occurring after a brief course of steroids. The impact of corticosteroids on CAM is complex. Firstly, they can induce immunosuppression by hampering migration, phagocytosis, and phagolysosome formation in macrophages. Secondly, corticosteroids can elevate blood glucose levels, exacerbating glycemic control in diabetic patients. Moreover, in countries like India, where corticosteroids are available over the counter, improper and prolonged steroid use might heighten susceptibility to mucormycosis [13,14].

The prevalence of osteomyelitis has notably increased in the year 2022 in comparison to 2021 followed by a gradual drop in 2023. In a previous study conducted by Kremers et al., it was found that the rate of osteomyelitis increased over the years, escalating from 11.4 cases per 100,000 people per year to 24.4 cases per 100,000 people per year [15]. There can be many reasons contributing to the increase in osteomyelitis in 2022. The lockdown imposed in response to the COVID-19 outbreak led to a surge in junk food consumption, consequently increasing sugar intake. This rise in sugar consumption could have contributed to a higher prevalence of dental caries, ultimately increasing the risk of osteomyelitis. The delay in dental care also would have resulted in undiagnosed or untreated periodontal disease, increasing the risk of complications such as osteomyelitis. Individuals with COVID-19 may experience alterations in immune function, including impaired inflammatory responses and compromised immune surveillance. These changes could potentially exacerbate existing inflammatory conditions [14]. 

In our study, fibro-osseous lesions were the third most commonly reported following inflammatory and infectious conditions and malignancies invading the bone. The range of percentage of the fibro-osseous lesions reported in all three years was between 10 and 20% of all bone lesions. In a previous series by Prabhu et al., fibro-osseous lesions contributed to 2% of all the overall cases. Similar to their results, ossifying fibroma was the most commonly reported fibro-osseous lesion in our study. In a systematic review by Kalmegh et al., it was found that fibrous dysplasia contributes to 2.5% of all bone tumors and 7% of benign bone tumors, stemming from congenital, metabolic, and genetic anomalies [15-17]. Another study by Akinyamoju et al. aimed to assess the utility of a predictive histologic index in identifying malignant changes in jaw fibro-osseous lesions, utilizing a retrospective analysis of hematoxylin and eosin (H&E) slides and archival records of fibrous dysplasia and ossifying fibroma cases. Results revealed varying degrees of malignancy, with a significant proportion of cases exhibiting moderate to low malignancy grades, suggesting the potential for malignant transformation and emphasizing the importance of further follow-up studies [18,19].

There were six (2.47%) reported cases of necrotic bone throughout all three years. The causative agent for five (83%) of the six cases was radiation. These patients had a history of radiotherapy and post-surgical treatment of oral cancer. A single case of medication-related osteonecrosis of jaw (MRONJ) was reported in 2023. The patient had a history of chemotherapy and was under bisphosphonates and denosumab, which are the most common medications that lead to MRONJ. 

Bone neoplasms were among the least two categories of our study. The highest percentage (6%) of benign pathology was reported during the year 2021, compared to the years 2022 (2.6%) and 2023 (3.07%). A constant increase in the primary malignancies invading the bone was also noted in our study. Specifically, 43% of the reported cases of bone pathologies involved malignancies involving the bone, especially squamous cell carcinoma. Metzger et al. found a significant increase in the tumor size of oral cancer as a result of the delay in treatment during the pandemic. Nath et al. showed similar findings, where a significant association was obtained between COVID-19 occurrence and oral cancer [20,21]. The restrictions and lockdowns during this period interfered with routine medical care and screenings. As our institution's exclusive oncology center was working with strict protocols even during the lockdown, there was a spike in the cases of patients who reported malignancies, considering the lower waiting period. The pandemic has also led to a notable increase in certain risk factors for the progression of oral cancer. These include increased use of alcohol and tobacco products, poor eating practices, high rates of obesity, and poor dental hygiene. Further, coronavirus infection impacts the immune system, leading to dysregulation of immune responses [22].

Identification of high-risk populations, elucidating underlying risk elements, and implementing targeted strategies for early identification and prevention all hinge on grasping the frequency of bone conditions in the head and neck. Various patterns of bone disease occurrence, including differences among age groups, genders, socioeconomic levels, and geographic regions, by rigorously and methodically analyzing and interpreting data is achieved. This will aid in comprehensive awareness of epidemiological trends which facilitates the development of focused interventions and prophylactic actions intended to reduce the prevalence of bone diseases and enhance public health results. The prevalence and impact of head and neck bone pathologies underscore the necessity for a comprehensive understanding of their etiology, pathophysiology, and clinical manifestations. Recognizing the signs and symptoms of these conditions is pivotal in facilitating early diagnosis and intervention, thereby mitigating potential complications and improving patient outcomes [22,23].

In summary, the three-year trend data points to a progressive decrease in the percentage of documented bone pathologies, which may reflect changes in disease patterns or diagnostic procedures. This study only involved data from the past three years in our institution. Using a larger dataset from multiple institutions over a wider duration will be vital. Clinico-demographical and pathological correlations will also be fruitful in generalizing the validity of the observed prevalence trends in bone diseases.

## Conclusions

These variations highlight the unpredictability of various bone pathologies and highlight the value of continuous observation and analysis to comprehend changing patterns in bone health. In the management of bone pathology, ongoing research and surveillance are crucial for identifying new trends, guiding clinical judgment, and enhancing patient outcomes. Additional research involving diverse populations and enhanced clinicopathological correlation is warranted in future investigations. This will provide deeper insights into the variations of bone pathologies across different demographic groups, leading to a more comprehensive understanding of disease mechanisms and improved clinical management strategies.
